# Detection of Infantile Movement Disorders in Video Data Using Deformable Part-Based Model

**DOI:** 10.3390/s18103202

**Published:** 2018-09-21

**Authors:** Muhammad Hassan Khan, Manuel Schneider, Muhammad Shahid Farid, Marcin Grzegorzek

**Affiliations:** 1 Research Group for Pattern Recognition, University of Siegen, 57076 Siegen, Germany; manuel.schneider@student.uni-siegen.de (M.S.); marcin.grzegorzek@uni-siegen.de (M.G.); 2 College of Information Technology, University of the Punjab, 54000 Lahore, Pakistan; shahid@pucit.edu.pk

**Keywords:** movement analysis, infantile movement disorders, part-based model, k-means clustering

## Abstract

Movement analysis of infants’ body parts is momentous for the early detection of various movement disorders such as cerebral palsy. Most existing techniques are either marker-based or use wearable sensors to analyze the movement disorders. Such techniques work well for adults, however they are not effective for infants as wearing such sensors or markers may cause discomfort to them, affecting their natural movements. This paper presents a method to help the clinicians for the early detection of movement disorders in infants. The proposed method is marker-less and does not use any wearable sensors which makes it ideal for the analysis of body parts movement in infants. The algorithm is based on the deformable part-based model to detect the body parts and track them in the subsequent frames of the video to encode the motion information. The proposed algorithm learns a model using a set of part filters and spatial relations between the body parts. In particular, it forms a mixture of part-filters for each body part to determine its orientation which is used to detect the parts and analyze their movements by tracking them in the temporal direction. The model is represented using a tree-structured graph and the learning process is carried out using the structured support vector machine. The proposed framework will assist the clinicians and the general practitioners in the early detection of infantile movement disorders. The performance evaluation of the proposed method is carried out on a large dataset and the results compared with the existing techniques demonstrate its effectiveness.

## 1. Introduction

Normal human movements, such as, moving an arm, look simple but require a complex coordination of control between the brain and the musculoskeletal system. Any disruption in the coordination system may result in inhabit unwanted movements, trouble in making the intended movements or both [1]. These may appear due to abnormal development of the brain, injury in the brain of a child during the pregnancy or at birth, or genetic disorders. Studies e.g., [2,3,4,5,6], have shown that the early detection of movement disorders plays an important role in the early intervention and establishing a therapy program for the recovery. To diagnose the movement disorders, the spontaneous movements of an infant are observed by the doctors or the physiotherapists along with the family medical history. This examination is known as the *general movement assessment* (GMA) [2,7]. However, it is a subjective procedure based on observer’s expertise and does not have any standardized criteria to measure the outcomes. Moreover, it is time consuming procedure to manually analyze every infant, therefore an automatic system is needed to accurately analyze the movements in various body parts of infant.

In recent years, numerous computer-based techniques have been proposed to assess the infant’s motion information and to perform quantitative analysis, using various techniques like movement tracking [8,9,10,11,12,13,14]. Some of them used wearable motion sensors [15,16,17] while the others used markers [4,18] on the infant’s body parts and tracked them using the visual sensors to encode the motion information. However, wearing a large number of sensors or markers may cause discomfort to the young patients [19] which may affect their natural body part movements. Several other techniques e.g., [20,21,22] have exploited the Microsoft Kinect to analyze the movement patterns in human body. They either use the Kinect’s integrated body tracking information [22] or body part model fitting technique [23] on depth images to encode the motion information of human body parts by detecting and tracking them in the subsequent frames of a video. Although the invention of Kinect sensor has triggered a lot of research on human motion analysis, rehabilitation, and clinical assessment; its limitations—that the subject must be in standing position and has size greater than one meter for body tracking—make it unsuitable for infants [21]. A review to analyze the movement disorders in infants using their spontaneous movements is presented in [24], and literature related to postural control and efficiency of movement can be found in [25,26,27,28,29].

This paper presents a novel computer vision-based method to detect and track infant’s body parts in video. The proposed method is marker-less and does not use any wearable sensors which makes it ideal for use in infants. It is based on the principle of pictorial structure framework [30,31,32] in which an object’s appearance is represented using a collection of part-templates (also called part-filters) together with the spatial relations between the parts. That is, the human body structure is detected in the proposed technique using the part-templates (part-filters) along with the spatial relations between the parts. In particular, the proposed method begins by detecting the different body parts which are based on joints’ locations, and computes angles at predicted joints such as elbow, shoulder, etc. Later, the movement at different joints is encoded by tracking the angle orientations in the temporal direction. To automatically detect the body parts, the proposed technique learns a model from a set of training images by preparing a set of part-filters and the spatial relations between the parts. Moreover, to handle the problem of appearance changes due to large articulation in human body parts, their appearance-changes are encoded using the mixture of part-filters for the respective part and the spatial relations between the involved parts. The model is represented using a tree-structured graph and the learning process is carried out using the structured support vector machine (structSVM). We recorded a dataset of 10 patients suffering from movement disorders in a local children hospital. The patients were being treated by the therapists in the recording days. The performance of the proposed algorithm is evaluated using two challenging ways: joints estimation accuracy and motion encoding accuracy. The results are compared with exiting state-of-the-art techniques which reveal that the proposed algorithm is very effective in analyzing the infantile movement disorders. The proposed approach is influenced by [31,32] and presents the following novel contributions:

- The proposed algorithm does not require wearing markers and other wearable sensors which makes it ideal for movement analysis of infants;
- The proposed technique performs movement analysis in videos by computing the angle orientations at different predicted joints’ locations and tracking them in the temporal direction;
- The proposal of a simple yet novel modeling of part-templates to deal with the self-occlusion of body parts and the rotation problems;
- A novel scoring scheme is introduced to eliminate the false positives in the detection of body parts;
- To deal with the vast variability in the different body parts, an optimal mixture size is chosen for each part to improve the detection process.
- A detailed review of the state-of-the-art techniques to encode the human body parts movement. The techniques are also classified into various categories based on their underlying body parts detection and motion encoding methods.

## 2. Related Work

**Figure 1:**
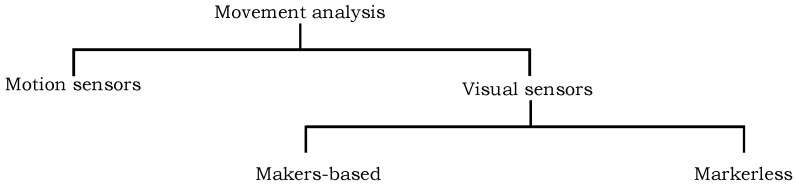
The distribution of existing techniques for movement analysis of human body parts into various categories.

Over the last few years, numerous computer-based techniques have been proposed to analyze the movement of human body parts for the clinical and behavioral assessment and other applications [33,34,35,36]. These techniques can be categorized in two groups: visual sensor-based approaches and motion sensor-based approaches. The first group of approaches either use markers on human body region or exploit markerless solutions by incorporating the image features such as color, edges, etc. to detect and track the different body parts in video data. The second family of approaches use motion sensors e.g., inertial measurement unit (IMU) to encode the motion information. Figure 1 presents the categorization of existing techniques into different groups which are reviewed in the following subsections.

### 2.1. Visual Sensor-Based Approaches

These techniques use color images, depth information or both for movement analysis. The methods e.g., [37,38,39] attach markers on human body to represent the joints’ locations, and use them to detect and track human skeleton in a video to compute the motion information, whereas the others exploit image features such as color, shape, and edges to estimate the joints’ locations for movement analysis.

#### 2.1.1. Marker-Based Techniques

The marker-based approaches use a set of markers e.g., infrared (IR) markers, reflective spheres, light-emitting diodes at human body, particularly, at joints’ locations to track the motion information. Tao et al. [40] proposed a color marker-based tracking algorithm to estimate the motion information at different joints. They attached different color markers on joints’ locations and tracked them in the video. The algorithm proposed in [4] encodes the motion of markers in 3D (three dimensional) domain using seven cameras. The markers are attached to the human body and their motion information is used to predict the risk for developing movement disorders. Burke et al. [37] proposed two games for upper limb stroke rehabilitation which are controlled by color object segmentation and its tracking for motion detection. They attach the color markers at the upper limbs and their method detects and tracks them using the calibration process. The system proposed in [38] uses color markers at the foot of the adults to analyze the foot positioning and orientation for gait training. The authors in [41] encodes the human motion information using a tiny high resolution video camera and IR-based markers.

Rado et al. [42] proposed an IR optical tracker-based motion tracking of the knees for patient rehabilitation. Based on the computed motion information, they detect the errors in movement and system demonstrates to the user how to perform the movement correctly. Chen et al. [39] developed a therapy system using an IR camera with hand skateboard training device for upper limb stroke rehabilitation. Patients participating in the therapy have a binding band attached to a hand skateboard on the table to guide the patient in moving the hand skateboard along the designated path. A recent survey on the evaluation of marker based system is presented in [43]. Since these markers have to hook-up with human body parts, they require cumbersome installation and calibration process [44]. Moreover, increasing the number of markers (i.e., hooking-up with each body part of infant) will raise the complexity in the tracking when markers are either close to each other or become occluded due to the size of infant’s body region. Additionally, increasing the IR-based markers makes the system expensive too.

#### 2.1.2. Markerless Techniques

Lately, the markerless techniques have gained attraction in the research community due to its several computer-vision-based applications [45,46,47,48]. Instead of using several markers at human body parts, they use image features such as shape, edges, and pixels’ location to detect and track different body parts. The method proposed in [49] predicts the 3D positions of human joints in depth image. It employs per pixel classification of human body parts using random forest classifier. Hesse et al. [20] proposed an improved version of [49] by exploiting random ferns to estimate the infant’s body parts using pixel-wise body part classification. They tracked angle orientation at predicted joints in the successive frames to encode the motion information. However, it is very difficult to classify the different body parts in depth images particularly when they are mixed-up with the other parts [20]. In [5,50], the authors proposed the computation of optical flow to estimate the movement patterns in the infant’s limbs. However, such techniques are unable to localize the movements at a particular joint. Evet et al. [51] developed a game for stroke rehabilitation. They captured the movement and gesture of hand using an optical camera and a thermal camera. The technique, however, is limited to recognize only two gestures: hand open and closed. A few techniques e.g., [21,52,53,54] use body part model fitting technique which comprises of basic shapes on depth images to detect the infant’s body parts and encode their movements. The accuracy of such techniques significantly degrades when the desired body parts are occluded [23].

In recent years, the Microsoft Kinect sensor is considered an effective and low cost device in the clinical assessment and the rehabilitation places to provide a markerless motion capturing system [21]. It consists of a visual and a depth sensor which enables to create a 3D view of the environment. Additionally, the depth sensor of Kinect provides the skeleton tracking of human which has been exploited by the several researchers at ambient assisted living and therapeutic places to analyze movements in humans. For example, the authors in [22] proposed a system to encode the motion information of a patient using the integrated skeleton information of human from the Kinect. They compare and evaluate the patient movements with the desired exercise and generate the feedback on screen. Guerrero et al. [55] used the Kinect skeleton information to estimate the patient’s posture and compared it with a model posture. These postures are required in some physical exercises to strengthen the body muscles. The technique proposed in [56] calculates the 3D coordinates distances between 15 joints by employing the skeleton information obtained from Kinect, and uses them to monitor the rehabilitation progress. Chang et al. [57] use the human skeleton tracking information from Kinect and proposed a rehabilitation system to assist the therapists in their work. The system is designed for children suffering from motor disabilities and presents the rehabilitation progress to the therapists, as per defined standards. The researches [58,59] exploits the Kinect’s skeleton tracking to analyze the rehabilitation in upper limbs. Chang et al. [59] also validate the tracking results of Kinect sensor using the output of motion capturing system, known as OptiTrack. Recent survey of different applications which encode the movement information of human body parts using Kinect can be found in [60]. Although the Kinect sensor provides real-time skeleton tracking of human with quite a good accuracy, its limitations—that the subject must be in standing position and has size greater than one meter for body tracking—prevent the automatic detection and movement analysis of infants.

### 2.2. Motion Sensor-Based Algorithms

Motion sensors e.g., accelerometers, gyroscopes, and magnetometers, are another mean to encode human motion information. Heinze et al. [16] proposed a system to capture the movements of infants’ limbs using four accelerometers attached with the limbs. They use a decision tree algorithm to classify these movements into healthy and abnormal. The technique proposed in [61] uses tri-axial accelerometers on the chest, thigh, and shank of the working leg to assess the rehabilitation progress of a patient suffering with knee osteoarthritis by encoding the motion information at the respective body parts. The authors in [62] proposed a system using a set of accelerometers and compass to capture human motion for home rehabilitation. The sensors are attached on specified movable body parts and the system assigns a score based on the quality of movement defined by the therapists. Chen et al. [63] developed a motion monitoring system using a set of wireless accelerometer sensors attached with the patient’s body parts to remotely monitor his/her movements. The movement information is shared with the clinic too using a web-based system. The technique in [64] proposed a system to assess the motor ability of stroke patients using four accelerometers, attached on the human upper limbs and on the chest. The motion information at these parts are extracted within each time segment for linear regression to predict the clinical scores of motor abilities.

Zhang et al. [65] proposed a wireless human motion monitoring system for gait analysis in rehabilitation process using a set inertial measurement units (IMU) and a pair of smart shoes with pressure sensors to measure the force distributions between the two feet during the walk. An IMU is the combination of gyroscopes, accelerometers, and magnetometers, which provides the motion information relevant to angular velocity and acceleration in the sensor/body and magnetic field around it, respectively. The authors in [66,67] compute the motion information from a set of IMUs to monitor the gait. Instead of hooking-up a set of individual sensors, the authors in [68] proposed a sensing jacket consisting of 10 IMUs for home based exercise trainer system. Later, the encoded information is compared with the desired exercise. Similarly, a smart garment is proposed in [69] using a set of IMUs to support posture correction. The systems alerts the user by vibrating on the garment and visual instructions on smartphone using bluetooth connection. The authors in [70] proposed the integration of Kalman filtering with inertial sensors to improve the overall estimation of human motion.

In [71], an electrogoniometer (an electric device to measure the angles at joints) is used to capture the motion information at different joints of children suffering from CP during the exercise. They employed such a motion information in a virtual reality (VR)-based game and claim that patients have shown great interest, performed more repetitions of the exercise, and generated more ankle dorsiflexion in comparison with standalone exercise. Although, VR-based gaming applications offer an interactive, engaging, and effective environment for physical therapy, they require expensive hardware and software setup. Moreover, they are designed to suit a specific class of patients and could not be useful in case of young patients as they cannot interact with such systems. In short, the limitation of wearable sensor-based motion detection techniques is that they require to wear several sensors on the human body which may cause discomfort to them (particularly for young patients) and may affect their natural movements [21].

## 3. Proposed Infant’s Movement Analysis Algorithm

**Figure 2:**
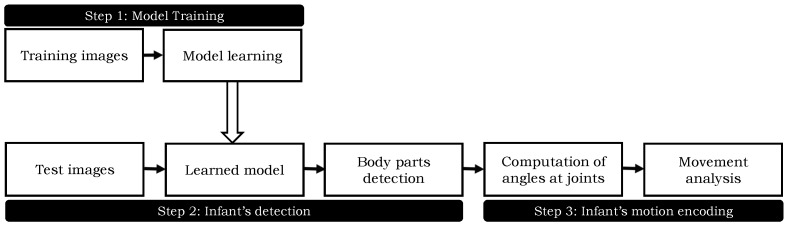
An overview of the proposed method.

The proposed algorithm works in three steps. First, a human body model is prepared to detect the skeleton of the infants. It generates a mixture of part-filters for each body part and encodes the spatial relations between them. Second, the body parts are detected in a given image using the trained model. Third, the angles are computed on different predicted joints’ locations and the motion information is recorded temporally. A block diagram of the proposed method is shown in Figure 2. The detail of each step is described in the following sections. To enhance the readability, the detection step is explained prior to the training of the model.

### 3.1. Proposed Template-Based Model for Infant’s Detection

**Figure 3:**
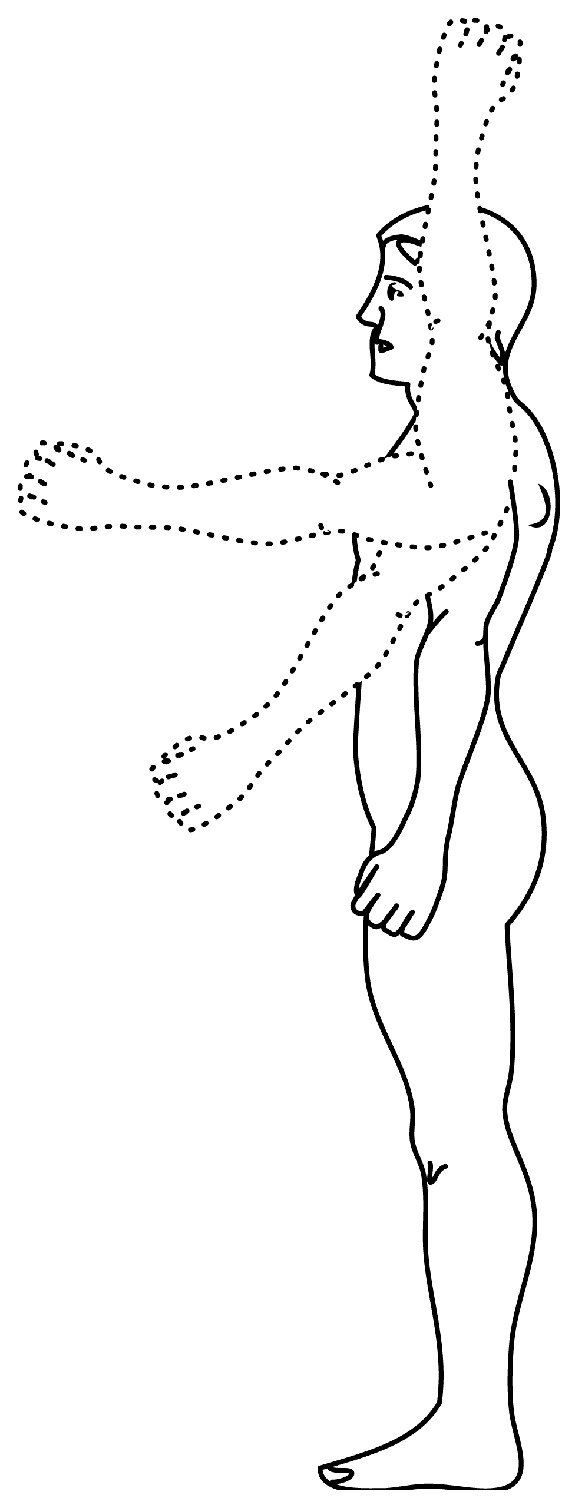
An example image illustrating the variations in the appearance of human arm due to large articulation [72].

Automatic detection of human body parts in an image is a challenging task due to the variations in their appearances because of colors, shapes, sizes, occlusions etc. Moreover, the human body has many degrees of freedom in the articulation of body parts which may result in extensive variations in their appearances. For example, Figure 3 presents such appearance-changes in the human arm. To cope with these problems, the proposed model prepares a mixture of part-filters for each body part and defines the spatial relations between the body parts. A part-filter in the same mixture may correspond to different orientations, called the ‘state’ of the body part, for example, horizontal versus vertical alignment of the hand. For a given image, the detection of body parts is performed on all locations and scales using part-filters and a score for each part-filter is computed. This score represents the likelihood of occurrence of a particular state. The score is computed by applying the part-filters to convolve over a histogram of oriented gradients (HOG) of the test image. This exhaustive search is carried out only once for the first frame of the video. Since the camera is fixed during the recording and infants are not making rapid movements, the predicted location of infant in previous frame with the relaxation of a certain threshold pixels ($\Omega_{dist}$) in all the directions is employed to set the search-space in the succeeding frame. This search-space optimization is not only helpful in improving the detection accuracy but also decreases the computation cost.

Let $f_{p_{a}}^{s_{a}}$ be a template of size M×N defined for part $p_{a}$ in state $s_{a}$, where $s_{a}\in \left\{1,\cdots ,S\right\}$ represents the set of states and a∈{1,⋯,K} represents the part. Let FR be the part-filter response or score in HOG image $I_{\Phi }$ at location $l=I_{\Phi }\left(x,y\right)$. The response is computed by matching $f_{p_{a}}^{s_{a}}$ with $I_{\Phi }$:

(1) $$FR\left(f_{p_{a}}^{s_{a}},I_{\Phi },l\right)=\sum_{m=1}^{M}\sum_{n=1}^{N}f_{p_{a}}^{s_{a}}\left(m,n\right)I_{\Phi }\left(x+m,y+n\right)$$

The part-filter scores are computed in a multi-scale fashion, however to keep the discussion simple we describe the algorithm here using at full-scale. Equation (1) is indeed a cross-correlation measure; the highest positive value represents the best matching location of respective part-filter.

**Figure 4:**
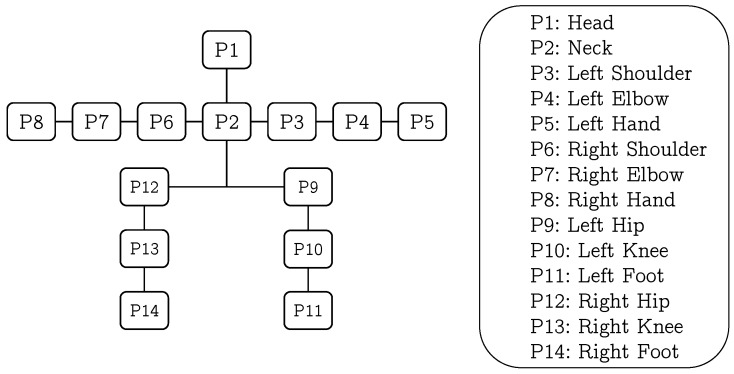
A kinematic tree of human body representing the relation between each body part.

The proposed model is represented using a tree-structured graph G=(V,E), where the set of vertices *V* represents the body parts (located at joints) and the edges *E* models the relations between them. A kinematic tree of human body representing the relation between the body parts is shown in Figure 4. To achieve the articulation between different parts, we used a set of ‘springs’ [31] to define a spatial relation between a part and its parent, e.g., hand and elbow. If we have 5 different states of a part and 5 different states of its parent, then there are 25 springs which define the relative placement of child and its parent part giving us 25 different orientations. Let $p_{a}$ and $p_{b}$ be a body-part and its parent-part respectively, the score for the detection of the part and its state is defined as:

(2) $$Score\left(I,l,s\right)=\sum_{a\in V}f_{p_{a}}^{s_{a}}\left(I_{\Phi },l_{a}\right)+D\left(p_{a},p_{b}\right)+CO\left(s\right)$$

Equation (2) consists of three terms. The first term is the part-filter response in $I_{\Phi }$ at location $l_{a}$. The second term *D* defines a spring model between part $p_{a}$ and $p_{b}$ using the distance information between them and can be described as:

(3) $$D\left(a,b\right)=\sum_{a,b\in E}\zeta_{p_{a},p_{b}}^{s_{a},s_{b}}\Psi \left(l_{a}-l_{b}\right)$$

where $\zeta_{p_{a},p_{b}}^{s_{a},s_{b}}$ is a deformation parameter which encodes the placement of a part relative to its rest location; i.e., the relative location of $p_{a}$ to its parent $p_{b}$ based on their states $s_{a}$ and $s_{b}$, respectively. The term Ψ,

(4) $$\Psi \left(p_{a}-p_{b}\right)={\left[d_{x},d_{y},d_{x}^{2},d_{y}^{2}\right]}^{⊤}$$

is the relative predictive displacement of $p_{a}$ with respect to $p_{b}$, where $d_{x}=x_{a}-x_{b}$ and $d_{y}=y_{a}-y_{b}$. Equation (3) computes the deformation cost, which describes the difference between the detected and presumed relative position of a part to its parent in xy-coordinates. In particular, it penalized the score in Equation (2) based on the deviation of predicted location from the rest location. The third term in Equation (2) describes the co-occurrences of parts’ states as in [32]:

(5) $$CO\left(s\right)=\sum_{a\in V}R_{p_{a}}^{s_{a}}+\sum_{a,b\in E}R_{p_{a},p_{b}}^{s_{a},s_{b}}$$

The first term in Equation (5) $R_{p_{a}}^{s_{a}}$ defines the assignment of one particular state for part $p_{a}$, while the pairwise feature $R_{p_{a},p_{b}}^{s_{a},s_{b}}$ represents a trained co-occurrence between the parts $p_{a}$ and $p_{b}$ using their states $s_{a}$ and $s_{b}$. As described earlier that the proposed algorithm uses a tree-structured graph *G* to define that which parts of the model have logical relations. It assign a positive score to the parts having a logical relation, and a negative score to the illogical relations.

The final score of a part $p_{a}$ is computed as the sum of local scores for all possible states $s_{a}$, achieved by maximizing Equation (2) over location *l* and states *s*. Let $z_{a}$ represents the pixel location and state of part $p_{a}$, that is $z_{a}=\left(l_{a},s_{a}\right)$. Let C{a} be children of part $p_{a}$. The score of part $p_{a}$ is computed as:

(6) $$S_{p_{a}}\left(z_{a}\right)=FR\left(f_{p_{a}}^{s_{a}},I_{\Phi },l\right)+\sum_{a\in V}R_{p_{a}}^{s_{a}}+\sum_{\overset{´}{a}\in C\left\{a\right\}}Score_{\overset{´}{a}}\left(z_{a}\right)$$

It can be noted from Equation (6) that the proposed model computes the local score of part $p_{a}$ at all pixel locations for the state $s_{a}$ by gathering the score from its children using Equation (2). The overall score is computed as,

(7) $$S_{b}\left(z_{b}\right)=\max_{z_{a}}\left[\begin{array}{c} S_{p_{a}}\left(z_{a}\right)+D\left(p_{a},p_{b}\right)+R_{p_{a},p_{b}}^{s_{a},s_{b}} \end{array}\right]$$

Score in Equation (7) is computed for part $p_{b}$ using the best scoring location and state of its child $p_{a}$. To efficiently search the entire human body structure, we exploited the concept of ”independence” assumption. For example, in a given torso instead of using many cascade loops for the detection of all other parts, the proposed method searches independently the best candidates of arm, leg, and so forth. Since we are using a tree-structured graph to encode the spatial relations between the parts, it can be achieved efficiently using dynamic programming [31]. In particular, the proposed method iterates over all the parts, computes the score starting from the leaf-node $p_{a}$ (i.e., foot) and passes this score to its parent part, and so forth. Eventually, this computation expands till the root part (i.e., head) by following Equations (6) and (7), and the high scoring root location determine the body-model. The proposed algorithm may introduce many overlapping detections in one image. Since the recorded data contains a single infant in each frame, we exploited non-maximum suppression and the highest scoring root location is greedily picked as an estimation of infant body. Moreover, the proposed model also maintains argmax indices in (7) (i.e., the location of a selected part) therefore, we can easily recreate the highest scoring model based solely on the root location. To deal with the self-occlusion of body parts which is common in case of infants’ movements, while saving the predicted parts’ locations in Equation (7) the proposed method also saves their respective detection scores. At retrieval, instead of just picking the highest scoring root configuration of parts, we iterate over each individual part and compare their scores with a pre-defined threshold $\Omega_{score}$. The maximum scoring location that satisfies the $\Omega_{score}$ is chosen as the correct part.

### 3.2. Movement Analysis

**Figure 5:**
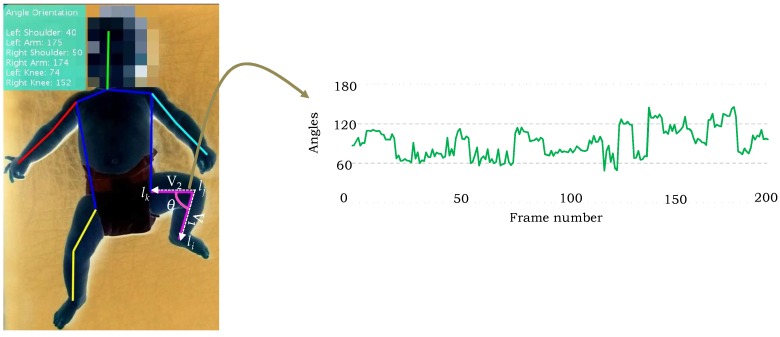
An example of angle computation at the predicted left knee joint and its tracking in the subsequent 200 frames. The angle orientations at other joints are annotated at the upper-left corner of the test image. The patient body is shown in negative to preserve the privacy of the subject.

The proposed method computes the angle orientation at the detected locations of body parts and encodes their tracking in the temporal direction to describe the motion information. Since the parts are located at joints, the skeleton information is extracted based on the predicted joints’ locations. The angles are computed at the joints and their tracking in the subsequent frames of the video instigates the movement in various parts, such as elbow, shoulder, knee, and etc. For example, consider the case of knee connected with the ankle and hip. Let $l_{i}$, $l_{j}$ and $l_{k}$ denote the ankle, knee and hip joints respectively (Figure 5), the following two vectors are computed:

(8) $$\begin{array}{c} V_{1}=l_{i}-l_{j}\\ V_{2}=l_{k}-l_{j} \end{array}$$

The angle θ between the vectors $V_{1}$ and $V_{2}$ representing the angle orientation at the knee joint is computed as,

(9) $$\theta =\arccos\left(\frac{V_{1}\cdot V_{2}}{|V_{1}\left|\right|V_{2}|}\right)$$

Analogously, the angles for other joints can be computed and tracked temporally in a video sequence to describe their respective movements. Figure 5 shows the angle orientations computed using the proposed algorithm on a sample image from the test dataset, and the tracking of angle at knee joint in the subsequent frames.

### 3.3. Model Training

**Figure 6:**
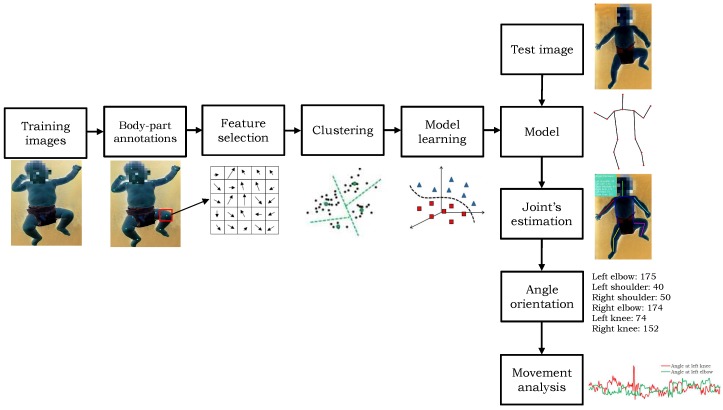
An illustration of model training, and the detection and the tracking of body parts. The patient body is shown in negative to preserve the privacy of the subject.

We used a set of positive images annotated with body parts’ locations, and a set of negative images without any human to train a model. Each positive image in the training dataset requires 14 annotated parts’ locations, as shown in Figure 6. The edge relations *E* in the tree-structured graph are defined manually by connecting the joints. To make the model robust and scale-invariant, the images are scaled, flipped, and rotated by a few degrees. For each annotated location of body part, the features are computed within the bounding box around the annotated part (Figure 6). To define the size of bounding box, the ratio between the length of each part in a given image to the median value of the length of respective part in the whole training set is computed, and then 75 percent quantile of the data (i.e., length) is selected to set the size of the bounding-box for all parts in that image. We computed HOG features in each bounding box to encode the appearance of the respective part and their orientation information is saved in 5×5 cells. Since it can be observe that the appearance changes in several parts (i.e., state) are based on the relative location of a part with respect to its parent. Therefore, the relative location of part with respect to its parent in all the training images are grouped into *S* clusters using the k-means clustering algorithm to define the all possible states of a part. We assumed that each cluster describes a unique state of the part. Moreover, we have not fixed the cluster size for each part, rather it is varying based on the degree of articulation in the part. For example, the arm and the leg parts comprises more articulation in comparison with the torso.

Within the aforementioned described scenario, our goal is to obtain a set of templates for each body part and the spatial relations between them such that the model assigns a high positive score to the predicted parts in the positive image and a low score to the parts in the negative image. More precisely, in a given training set of positive images {$I_{pos},l_{pos},s_{pos}$} and negative images {$I_{neg}$}, the learning of model parameters consists of finding a set of part-filters and deformation parameters which are computed using the structured prediction objective function proposed in [31]. Let’s assume that $z_{n}\;=\;\left(l_{n},s_{n}\right)$ and β is the set of part-filters and the relations between them, then using Equation (2) we can write $S\left(I,z\right)=\beta .\left(I_{\Phi },z\right)$. In particular, these parameters should satisfy the following two constraints:

(10) $$\begin{array}{c} \beta .\left(I_{pos},z\right)\geq +1\;\forall I_{pos}\\ \beta .\left(I_{neg},z\right)\leq -1\;\forall I_{neg} \end{array}$$

To learn the model parameters, the following optimization problem is solved:

$$\begin{array}{c} \arg\;\min_{\beta }\;\frac{1}{2}∥\beta {∥}^{2}+C\left(\sum_{pos}\xi_{pos}+\sum_{neg}\xi_{neg}\right)\\ s.t.\;\beta \cdot \left(I_{pos},z\right)\geq 1-\xi_{pos},\;\xi_{pos}\geq 0,\forall I_{pos}\\ \;\;\beta \cdot \left(I_{neg},z\right)\leq -1+\xi_{neg},\;\xi_{neg}\geq 0,\forall I_{neg}, \end{array}$$

where $\xi_{pos}$ and $\xi_{neg}$ are the slack variables representing the loss functions for positive and negative images respectively, and *C* is a user-defined regularization parameter which plays an important role in maximizing the margin and minimizing the loss function. To learn a set of part-filters and their spatial relations, structSVM [73] is an optimal solution however, we used an extension of structSVM proposed in [74]. Similar to the liblinear SVM [75], it uses a dual coordinate descent technique to find an optimal solution in a single pass. The required change in the above derivation is the ability of linear constraints that it should share the same slack variable for all the negative examples belonging to the same image $I_{neg}$ and solve the dual problem coordinate-wise by considering one variable at a time. The above formulation can be described as,

(11) $$\begin{array}{c} \arg\;\min_{\beta ,\xi_{n}\geq 0}\frac{1}{2}∥\beta {∥}^{2}+C\sum_{n}\xi_{n}\\ s.t.\;\beta \cdot \left(I_{n},z\right)\geq 1-\xi_{n},\forall_{n}\in pos\\ \;\;\beta \cdot \left(I_{n},z\right)\leq -1+\xi_{n},\forall_{n}\in neg. \end{array}$$

## 4. Experiments and Results

### 4.1. Evaluation Dataset

**Figure 7:**
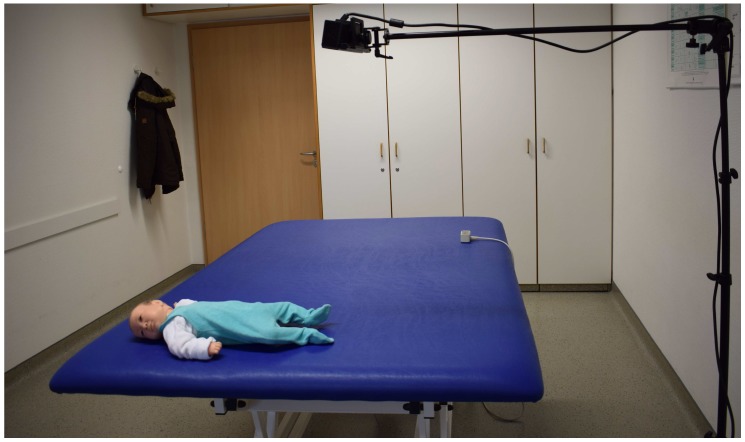
Camera setup in dataset acquisition.

To the best of our knowledge, there is no public dataset available to analyze the movement disorders in infants. Therefore, we captured a dataset in local children hospital using Microsoft Kinect. It is worth mentioning that we used only RGB data from Kinect in the proposed algorithm therefore, any other simple camera can be used too. We selected 10 patients of ages 2 weeks to 6 months with both genders, having movement disorders and currently they are being treated by the therapists. The informed consent was obtained from all participating individuals, the therapists and the infants’ parents. The camera was fitted on a tripod at height of 1.5 m with an angle of 90${}^{\circ }$ from the table surface. Figure 7 shows the camera setup during used in the recording. The subjects were lying on a table in supine lying position (i.e., lying on back), wearing only diapers which helped us to clearly capture their movements. For each patient, the recording session usually lasted for 15 minutes. A total of 20 video sequences comprising more than 25,000 frames were captured and used in the experiments. The ground truth of the test dataset was obtained through manual delineation. A team of three members carefully analyzed each case and manually marked the positions of the joints in each test image.

### 4.2. Experimental Setup

**Figure 8:**
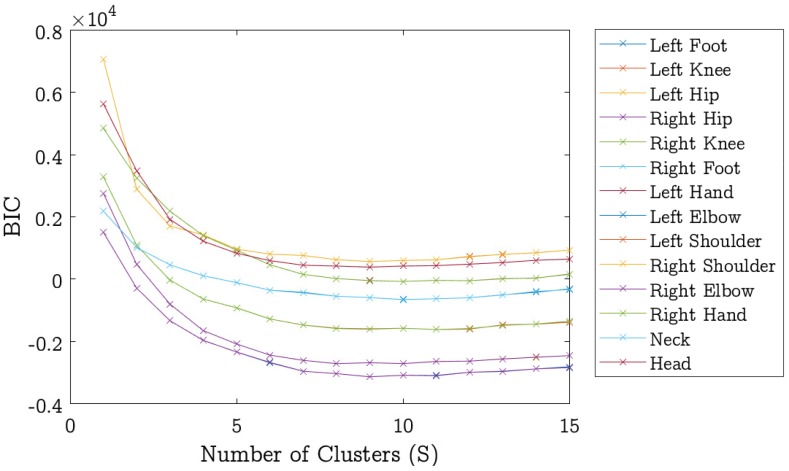
The number of clusters predicted by the Bayesian information criterion (BIC) for each body-part. A few plots are very close to each other and therefore they largely overlap and are not visible at this scale.

To train the model, we selected 650 positive images. All positive images were flipped and rotated between −15${}^{\circ }$ to +15${}^{\circ }$ with the interval of 5${}^{\circ }$ to obtain a robust model. This helps the model to learn the various states of the body parts. As described earlier that each positive image has 14 annotated locations of body parts, we computed HOG features in a bounding box around the marked location to encode the appearance of the respective body part. To describe the states of a particular part, the relative locations of that part with respective parent part in all images are clustered. The cluster size *S* depends on the degree of variation in the body part. The parts with large variety of appearances require more part-filters for accurate detection. We used the Bayesian information criterion (BIC) [76] to estimate the cluster size *S* for each body part. The BIC also known as Schwarz criterion is a criterion designed to select an optimal model among a finite set of models. To represent the different appearances of a part, we use k-means clustering with *S* number of clusters. The BIC value is computed as in [77]:

(12) $$\mathrm{BIC}=n\cdot \ln\left(\frac{R}{n}\right)+k\cdot \ln\left(n\right)$$

where *n* is the size of the data, *R* is the residual sum of squares, and *k* represents the number of model parameters. The parameter k=S(d+1), where *S* as the number of clusters and *d* is the dimension of data. The BIC value is computed for each body-part at varying the number of clusters *S* and the value of *S* that gives the minimum BIC value is chosen as the optimal number of clusters for that body-part. A plot of BIC values and clusters for all body-parts is shown in Figure 8. The optimal number of clusters found though BIC approach are listed in Table 1. We also experimentally tested different cluster sizes for each body part and listed the optimal sizes in Table 1. It can be noted that for right and left elbow parts, both empirical and BIC predicted cluster sizes are same. In other body parts, the empirically estimated cluster size is 2–3 cluster smaller than the BIC predicted size, except the head where the difference is 5. We observed that the performance of the proposed algorithm with BIC predicted clusters is almost same as when empirically estimated cluster sizes are used. Increasing the number of clusters does not improve the accuracy, it instead adversely affects the computational time of the algorithm. From experiments we found that using the BIC predicted cluster sizes, the computational time of the proposed method doubles without significant improvement in the detection accuracy. Nevertheless, using BIC one can automate the cluster size estimation and this can certainly save the time spent in experimentally estimating the cluster size for each body part.

We also input negative images to the model, the images with no human subjects. Each of the possible root locations in a negative image represents a unique negative example in the training set. We initialized the deformation parameters with [0,0,0.01,0.01] which demonstrates that the part location is close to its rest location. The structSVM library [74] is used in the implementation of the proposed method. Before the actual model is trained on full training database, a 10-fold cross validation is performed to validate the model by selecting the optimal value of its parameter *C*. We used multi-resolution search to find the optimal value of the hyper-parameter. That is, first, the parameter values are tested from a larger range and the best configurations is selected. Then a narrow search space is exploited around this value to select the optimal value in the second step.

### 4.3. Results

The trained model is evaluated on a probe (i.e., testing) dataset comprising the rest of the recordings of infants. Since the body parts are located on joints and the accuracy of encoding the motion information is also based on the precision of predicting the joints’ locations, the performance of the proposed algorithm is evaluated using the estimation of joints and the encoding of motion at particular joints. The short description of the results in each category is summarized in the subsequent sections.

#### 4.3.1. Joints Estimation Accuracy

The performance of the proposed algorithm in estimating the joints’ locations is assessed using two challenging matrices: Average Joint Position Error and Wost Case Accuracy. The results are compared with the recent existing similar techniques [20,78]. Furthermore, the encoded movement at different predicted joints are also compared with the manually annotated ground truth information. The Average Joint Position Error (AJPE) metric measures the average difference between the predicted locations of joints and their ground truth information. The average differences are measured in millimeters and the results are documented in Table 2. The results show that the proposed algorithm outperforms [20] in the detection of each body part. The mean AJPE of the method in [20] is 41, whereas our method has just over 12.

The Worst Case Accuracy (WCA) metric is defined as the percentage of frames in which all the joints must be detected within a certain threshold distance ($\Omega_{wca}$) from the ground truth information. It must be noted that any frame exhibiting error on even one joint location larger than $\Omega_{wca}$ would be considered as false positive. Similar to [78], we conducted two evaluations using $\Omega_{wca}$ 5 cm and 3 cm, and the results are summarized in Table 3. The results reveal that our method performs better in both tests achieving an accuracy of more than 95% and 86%, respectively.

#### 4.3.2. Motion Encoding Accuracy

**Figure 9:**
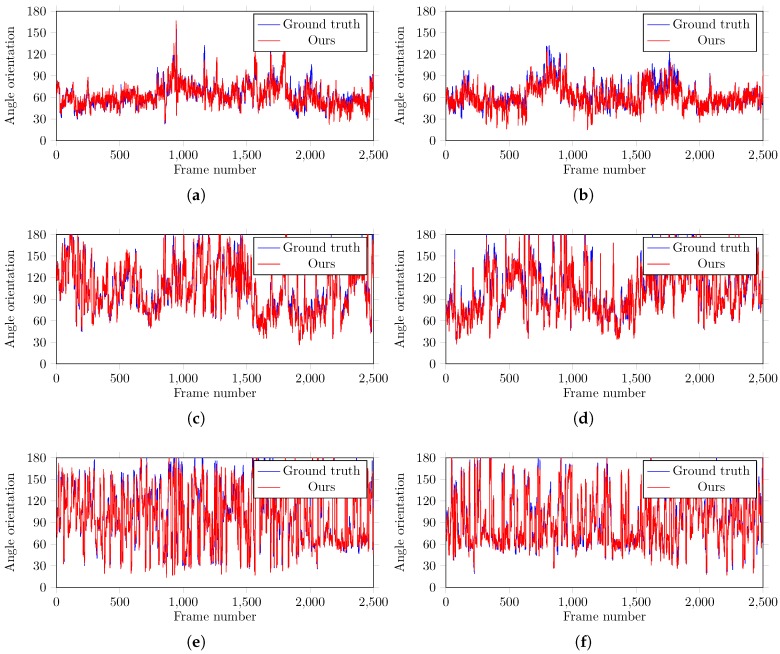
Predicted and the ground-truth angle orientations of (**a**) right shoulder, (**b**) left shoulder, (**c**) right elbow, (**d**) left elbow, (**e**) right knee, and (**f**) left knee in a test video sequence with 2500 frames.

We also evaluated the performance of the proposed algorithm in the encoding of motion information at predicted joints. The computed information is compared with the ground-truth information. In particular, we computed the angle orientation at the predicted joint locations, such as, shoulders, elbows, and knees. The orientation information is encoded in the temporal direction at particular joints. Figure 9 presents the computed movement information and the ground-truth angles. The overlapped areas represent similar movement patterns. Though there is little difference in the computed and the ground-truth movement patterns, one can observe that the estimated movement information (using angles) reflect the ground-truth accurately. The reason for the this small variation in results is that the body parts are detected a few pixels away from their actual locations (i.e., ground-truth information). In particular, the proposed algorithm estimates the joint’s location as the center of predicted body part patch in the image, and then draws edges between the estimated locations to calculate the angle orientation. Therefore, the deviation of part-filter window (i.e., detection results) by a few pixels from the ground-truth information of body part generates these small variations in computing the angles. Such plots (Figure 9) can help the doctors and the therapists to identify the movement disorders based on the absence of specific motion information at a particular joint.

To further investigate the performance of the proposed method, Mean Absolute Error (MAE) metric is also computed between the estimated movement information in terms of orientation angles and the ground-truth information. The results are presented in Table 4. The results reveal that the proposed algorithm is very accurate in computing the movement information with mean absolute error of around $3^{\circ }$ with respect to the ground-truth.

One can observe that the evaluation of the proposed method using different metrics, AJPE, WCA, and MAE perform consistently better in estimating the joints’ locations and encoding of motion information which reflect the efficacy of the proposed algorithm.

## 5. Conclusions and Future Work

In this paper, a novel method to identify the infantile movement disorders is presented. Unlike existing techniques, it does not use markers or sensors on the subject’s body to analyze their movements. A part-based model to detect and track the body parts of infants in video is proposed. The trained model encodes the possible orientations of each part and the spatial relations between the parts. In the probe sequence, the predicted joint locations are used to construct a skeleton and compute the angle orientations, and their tracking in the subsequent frames facilitates the movement analysis of a particular joint. In future, we plan to exploit the depth information to compute accurate angle orientations in 3D domain.

A few interesting applications of the proposed algorithm would be to evaluate the patient’s poses and movement during the therapeutic procedure. For example, motor disability is the special kind of disease arise in human due to a damage in the central nervous system (i.e., brain and spinal cord), associated with the body movements. It introduce several problems such as cerebral palsy, spinal scoliosis, peripheral paralysis of arms/legs, hip joint dysplasia and various myopathies [21]. To deal with such problems, the neurodevelopmental treatment and the Vojta techniques are the most common approaches [79]. The neurological physiotherapy aims to make available the message path between the brain and the musculoskeletal system by assisting the patients to perform blocked movement patterns. During the treatment, a particular stimulation is given to the patient body region to perform these blocked movement pattern which the patient is unable to perform in a normal way. The proposed method can be extended to detect the accurate poses and movements of the patient during the treatment, which ultimately reveals the accuracy of the given treatment. Since the therapist suggests an in-home continuation of the therapy in order to accomplish the best outcomes, an implementation of such a system may serve as in-home therapy alternative to in-hospital therapy. This would not only be helpful for the quick recovery of the patients but also useful for the patients who do not have access to the desired treatment in their towns.

Occlusions in some positions are unavoidable. If a body part is partially occluded, the proposed method is able to accurately detect the movement. However, in case of significant occlusions, increasing the number part filters (clusters) might not achieve the desired results, limiting the performance of our method. To deal with large occlusions, in future, we plan to extend the proposed algorithm using multiple cameras rather than using a single camera setup which would help to cater the large occlusions and to improve the accuracy of the algorithm.

## Figures and Tables

**Table 1:** The predicted number of clusters by the Bayesian information criterion (BIC) and BIC value for each body-part. ’Emp.’ represents the number of clusters obtained empirically for each body-part.

Body-Part	BIC Value (×$10^{3}$)	BIC Clusters	Emp. Clusters
Head	−0.67	9	4
Left Elbow	−1.63	9	9
Left Foot	−2.73	10	6
Left Hip	−2.73	8	6
Left Knee	−1.63	11	8
Left Shoulder	−0.67	9	6
Neck	−0.09	9	4
Right Elbow	−3.15	9	9
Right Foot	0.55	10	6
Right Hand	0.55	10	8
Right Hand	−3.15	10	8
Right Hip	−0.09	8	6
Right Knee	0.37	11	8
Right Shoulder	0.37	9	6

**Table 2:** Average joint position error measure the average difference (in millimeter) between the predicted and the ground truth locations. The error is reported per body part and Mean Error represents the average error across all the body parts in the test dataset. The proposed method outperforms in the detection of each body (i.e., having minimum error).

Body-Parts Detection Error		
Body-Part	Hesse et al. [20]	Proposed Method
Head	37	20.3
Neck	20	11.4
Right Shoulder	27	11
Left Shoulder	73	11.4
Right Elbow	24	11.2
Left Elbow	20	12.4
Right Hand	44	11.9
Left Hand	149	14.4
Right Hip	33	11.9
Left Hip	12	11.2
Right Knee	45	11.9
Left Knee	49	11.7
Right Foot	28	14
Left Foot	30	12.8
Mean Error	41	12.7

**Table 3:** Performance evaluation using WCA metric which describe the percentage number of frames in the test dataset where all the body-parts are detected within a certain threshold distance ($\Omega_{wca}$) from the ground truth location. Two experiments are carried out using $\Omega_{wca}$ = 5 cm and $\Omega_{wca}$ = 3 cm. The best results are marked in bold.

Method	$\Omega_{\mathrm{wca}}$ = 5 cm	$\Omega_{\mathrm{wca}}$ = 3 cm
Hesse et al. [78]	90.0%	85.0%
Proposed method	**95.8%**	**86.3%**

**Table 4:** Mean Absolute Error (MAE) in the estimated and the ground-truth angle orientations of different body parts.

Left			Right			Average
Elbow	Knee	Shoulder	Elbow	Knee	Shoulder		
3.632	2.959	2.830	3.231	3.160	2.438	3.042
